# Exposure of human intestinal epithelial cells and primary human hepatocytes to trypsin-like serine protease inhibitors with potential antiviral effect

**DOI:** 10.1080/14756366.2021.1886093

**Published:** 2021-03-01

**Authors:** Erzsébet Pászti-Gere, Judit Pomothy, Ákos Jerzsele, Oliver Pilgram, Torsten Steinmetzer

**Affiliations:** a Department of Pharmacology and Toxicology, University of Veterinary Medicine, Budapest, Hungary; b Faculty of Pharmacy, Institute of Pharmaceutical Chemistry, Philipps University Marburg, Marburg, Germany

**Keywords:** HIEC-6, PHH, trypsin-like serine protease inhibitors, occludin, H_2_O_2_

## Abstract

Human intestinal epithelial cell line-6 (HIEC-6) cells and primary human hepatocytes (PHHs) were treated with 3-amidinophenylalanine-derived inhibitors of trypsin-like serine proteases for 24 hours. It was proven that treatment with MI-1900 and MI-1907 was tolerated up to 50 μM in HIEC-6. These inhibitors did not cause elevations in extracellular H_2_O_2_ levels and in the concentrations of interleukin (IL)-6 and IL-8 and did not alter occludin distribution in HIEC-6. It was also found that MI-1900 and MI-1907 up to 50 μM did not affect cell viability, IL-6 and IL-8 and occludin levels of PHH. Based on our findings, these inhibitors could be safely applicable at 50 μM in HIEC-6 and in PHH; however, redox status was disturbed in case of PHH. Moreover, it has recently been demonstrated that MI-1900 prevents the replication and spread of the new SARS-CoV-2 in infected Calu-3 cells, most-likely via an inhibition of the membrane-bound host protease TMPRSS2.

## Introduction

There are approximately 70 different trypsin-like serine proteases in humans, which fulfil manifold physiological functions. Dysregulated activities of these proteases contribute to the development of numerous diseases, like thrombotic and bleeding disorders, cancer, iron metabolic diseases, pancreatitis, or osteoarthritis^1–9^. Despite these potential applications for drug development, so far, a broad application was only achieved with synthetic inhibitors of the blood clotting proteases thrombin and factor Xa have been approved for use as anticoagulants. A relatively non-specific scaffold of numerous trypsin-like serine protease inhibitors comprises tertiary amides of arylsulfonylated 3-amidinophenylalanines (Phe(3-Am)), like tosyl-Phe(3-Am)-piperidide (TAPAP)^10^. Mesupron, a structurally closely related hydroxyamidino-prodrug of the urokinase-type plasminogen activator inhibitor WX-UK1^11^ reached phase II development for cancer therapy. Several derivatives of this scaffold have been developed as inhibitors of other trypsin-like serine proteases including type II transmembrane serine proteases (TTSPs) like matriptase^12^^,^^13^. Related TTSPs, like transmembrane protease serine 2 (TMPRSS2) or human airway trypsin-like protease (HAT), and differentially expressed in squamous cell carcinoma-1 (DESC1) protein can also activate surface glycoproteins of different viruses, e.g. certain influenza viruses^14^. This activation step is essential for the fusion competence of these viruses and therefore a prerequisite for their replication and spread. TMPRSS2 is also involved in the activation of the spike (S) surface protein of various coronaviruses, including the new SARS-CoV-2^15^^,^^16^, as well as the activation of fusion proteins of several other respiratory viruses, e.g. human metapneumovirus or human parainfluenza viruses^14^^,^^17^.

Coronaviruses are a family of enveloped, single-stranded RNA viruses. The severe acute respiratory syndrome coronavirus (SARS-CoV) – and the Middle East respiratory syndrome coronavirus (MERS-CoV) can cause strongly pathogenic respiratory diseases. Four additional human pathogenic coronaviruses, human coronavirus-229E (HcoV-229E), HcoV-HKU1, HcoV-NL63, and HcoV-0C43 have been reported until now, but infections with these viruses only led to mild symptoms. In December 2019, a newly discovered type of coronavirus, the SARS-CoV-2 began to spread throughout the world from Wuhan, China, causing a pandemic disease, COVID-19. The development of potential drugs capable of targeting the S protein activation is of high interest.

Coronaviruses have evolved multiple strategies for proteolytic activation of the S-protein and depending on the virus strains, various host proteases like the trypsin-like serine protease TMPRSS2 or furin are involved. It has been proven that TMPRSS2 as a host cell factor is critical for the infectivity of several clinically relevant viruses including coronaviruses^18–23^. Tarnow et al.^24^ reported that H1N1 and H7N9 influenza virus replication was significantly suppressed in airway explants in TMPRSS2 deficient mice; however, knockout of TMPRSS2 expression in mice only exerted a minor effect on H3N2 virus replication. Therefore, additional trypsin-like serine proteases seem to be involved in the activation of H3N2 influenza A as well as influenza B viruses^25^.

Hoffmann et al.^15^ proved that host cell entry of SARS-CoV-2 depends on the SARS-CoV receptor angiotensin-converting enzyme 2 (ACE2) involving the cellular serine protease TMPRSS2 for S protein priming. The latter step can be blocked by camostat, a clinically proven inhibitor of numerous trypsin-like serine proteases. Treatment with camostat could exert partial inhibition of S-driven entry of SARS-CoV-2 into human colon adenocarcinoma Caco-2 cells and Vero-TMPRSS2 cells.

Bestle et al.^16^ demonstrated that both TMPRSS2 and furin are responsible for S activation of SARS-CoV-2 in human Calu-3 airway epithelial cells, but at different sites. It was shown that furin cleaves the S protein at its S1/S2 site and TMPRSS2 at the S2′ site. Moreover, Calu 3 cells inoculated with SARS-CoV-2 at low multiplicity of infection (MOI) showed only small foci of infection if they were previously exposed to antisense peptide-conjugated phosphorodiamidate morpholino oligomer (PPMO) leading to knockdown of TMPRSS2 activity. Based on these data, it was ascertained that TMPRSS2 as host cell factor is a prerequisite for SARS-CoV-2 activation and replication in Calu-3 cells and inhibition of TMPRSS2 activity could successfully block viral infectivity. A similar effect was found in vesicular stomatitis virus (VSV) pseudotype particles bearing the S protein of the SARS-CoV-2^26^. These results suggest that non-toxic and biochemically well characterised inhibitors of trypsin-like serine proteases, like TMPRSS2, could be potential drugs for the treatment of SARS-CoV-2 infections. Therefore, we characterised several inhibitors of the Phe(3-Am)-type in selected cells.

The human intestinal epithelial cell line-6 (HIEC-6) is a non-tumourigenic intestinal epithelial crypt cell line^27^ located in the small intestine. Epithelial cells play a major role in absorption and secretion processes in the gastrointestinal tract, as well as in protection from pathogens and xenobiotics via their intestinal barrier^28^. Therefore, intestinal epithelial cells (primary or cell lines) can be appropriate models for studying the pharmacokinetical effects of xenobiotics *in vitro*. The human colon adenocarcinoma cell line-2 (Caco-2) is a tumourigenic intestinal cell line, which is widely used for investigations of drug transport and paracellular effects^29^, although cancerous characteristics of Caco-2 limit its suitability for modelling physiological *in vivo* conditions.

In this study, sulfonylated Phe(3-Am)-derived inhibitors of trypsin-like serine proteases were applied on a non-tumourigenic human intestinal epithelial cell line, HIEC-6 and on primary human hepatocytes (PHHs) to estimate the safety of these potential antiviral agents *in vitro*. Cell viability assays were performed to determine non-cytotoxic concentrations of these inhibitors. Furthermore, redox balance and inflammatory status including production of interleukin (IL)-6 and IL-8 of the cells exposed to the inhibitors, MI-1900 and MI-1907 were elucidated in addition to assessment of subcellular distribution and quantity of the tight-junction (TJ) protein, occludin after inhibitor treatment. Furthermore, using a homology model of TMPRSS2, the binding mode of inhibitors MI-1900 and MI-1907 in complex with TMPRSS2 was modelled.

## Materials and methods

### Cell culture

The human intestinal epithelial cell line, HIEC-6 (ATCC, Manassas, VA) was grown in 50% Dulbecco’s Modified Eagle’s Medium (DMEM) and 50% Ham’s F12 Nutrient Mixture (Merck, Darmstadt, Germany) supplemented with 1.5 mmol/L HEPES, 1% insulin/transferrin/sodium selenite media supplement, 5 ng/mL epidermal growth factor, and 1% penicillin/streptomycin (all purchased from Invitrogen, Thermo Fisher Scientific, Waltham, MA). Cells were cultured on 96-well-plates for MTS assay and on 24-well transwell inserts (polyester, 0.4 µm pore size, Corning, Merck, Darmstadt, Germany) for the other procedures at 37 °C in a humidified atmosphere of 5% CO_2_. The complete culture medium was changed every two days until the cells reached the confluent condition.

Cryopreserved human primary hepatocytes were purchased from Thermo Fisher Scientific (Waltham, MA). Hepatocytes were seeded on membrane insert plates (Costar Transwell permeable supports, 0.4 µm polyester membrane 24 mm insert, six-well plate, tissue culture treated, Merck, Darmstadt, Germany) and on a 96-well plate (Merck, Darmstadt, Germany) for MTS assay. The seeding density was 0.9–1.1 × 10^6^ cells/mL, in 2 mL apical medium. The maintenance medium was Williams E medium, supplemented with 1% penicillin/streptomycin, 2 mM glutamine, 0.2 IU/mL insulin, and 0.22% bicarbonate (all purchased from Invitrogen, Thermo Fisher Scientific, Waltham, MA). Foetal bovine serum (FBS) 10% was added to the medium only in the first 6 h after thawing. The maintenance medium without FBS was then used and replaced every 24 h. The cells were incubated at 37 °C, with 5% CO_2_.

### Inhibitors MI-1900 and MI-1907

Inhibitors MI-1900 and MI-1907 were synthesised analogously to previously published inhibitors of this type^13^. Detailed synthesis procedures will be published elsewhere.

### Exposure of HIEC-6 and PHH to the synthetic inhibitors

**Figure 1.**
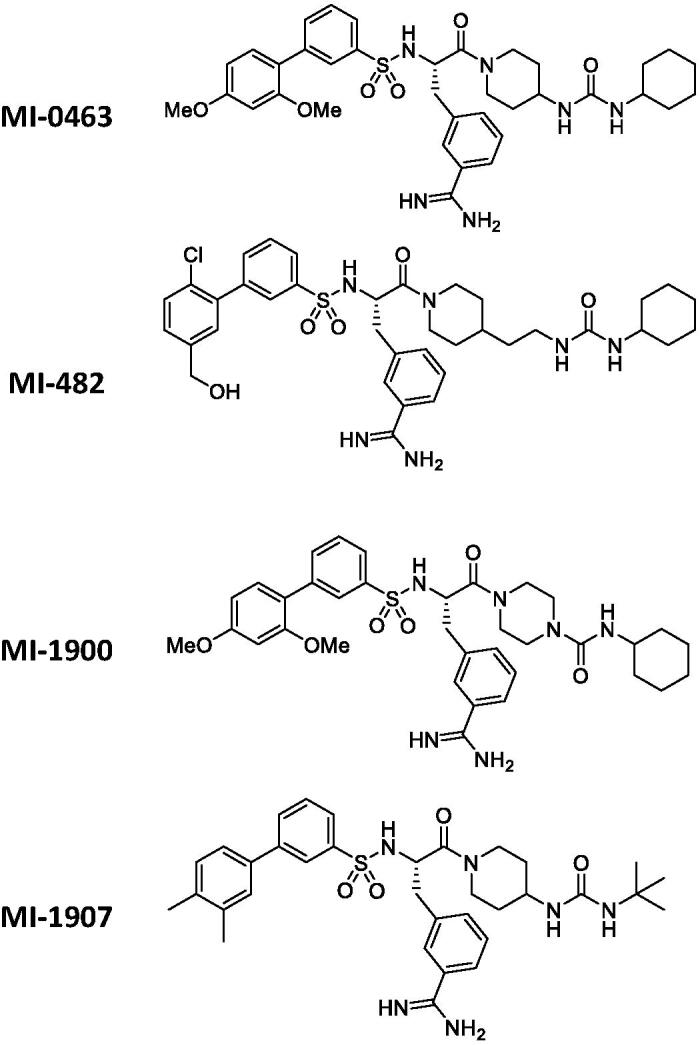
Structures of the used protease inhibitors.

For the experiments, four different Phe(3-Am)-derived inhibitors, MI-463, MI-482, MI-1900, and MI-1907 were used, and their structures are summarised in Figure 1. 10 mM stock solutions in dimethylsulphoxide (DMSO) were prepared and kept at −20 °C. Before treatment, confluent HIEC-6 and PHH were washed twice with plain (serum-free) medium. The solutions of the inhibitors further diluted in plain medium at 5, 20, 50, and 100 µM were prepared freshly from the stock solutions prior to each experiment. After incubation of the cells with the inhibitors for 24 h, the cells were washed twice with plain medium before being subjected to the subsequent procedures.

### MTS assay

The assay is based on the ability of living cells to reduce the MTS tetrazolium compound to a coloured formazan product soluble in the cell culture medium. The reduction is carried out by an NAD(P)H-dependent dehydrogenase in metabolically active cells. The HIEC-6 and the hepatocytes were placed onto 96-well-plates and incubated for 24 h with the inhibitors at 5, 20, 50, and 100 µM concentrations. The MTS assay was performed with eight parallels at each inhibitor concentration. After removing the medium and three times washing of the cells with PBS, 20 µL of CellTiter96 Aqueous One Solution (Promega Corporation, Madison, WI) containing MTS and an electron acceptor reagent, phenazine ethosulfate, were pipetted into a 96-well plate, each containing 100 µL of phenol red free medium. The plate was incubated for 1.5 h in a 5% CO_2_ incubator. The viability of HIEC-6 was detected with an EZ Read Biochrom 400 microplate reader (Biochrom, Cambridge, UK) at 490 nm.

### Extracellular H_2_O_2_ measurement by the Amplex Red method

The quantification of H_2_O_2_ concentrations in cell supernatants was carried out using the Amplex Red Hydrogen Peroxide Assay Kit (Invitrogen, Molecular Probes, Waltham, MA). In the presence of horse radish peroxidase, Amplex Red reagent reacts with H_2_O_2_ (in 1:1 stoichiometry) to produce a red fluorescent product called resorufin. Following the exposure of HIEC-6 and PHH to MI-1900 and MI-1907 (50 µM, 24 h), the H_2_O_2_ concentrations in the medium were determined using a working solution of 100 µM Amplex Red and 0.2 U/mL HRP. After 24 h, cell free supernatants were taken from the basolateral compartment. Fifty microlitres of the collected cell free supernatants were mixed with the Amplex Red working solutions. The fluorescence intensities were measured with a fluorometer (Victor X2 2030, Perkin Elmer, Waltham, MA) using 560 nm excitation and 590 nm emission wavelengths.

### Determination of pro-inflammatory cytokine expression

IL-6 and IL-8 concentrations were determined in HIEC-6 cell free supernatants and PHH supernatants using human IL-6 and IL-8 sandwich ELISA kits (Merck, Darmstadt, Germany). To elucidate the cytokine levels after 24 h treatment, the supernatants were treated according to the instructions of the manufacturer and absorbances were measured with an EZ Read Biochrom 400 microplate reader (Biochrom, Cambridge, UK) at 450 nm.

### Localisation of occludin distribution via immunofluorescent staining and evaluation of occludin concentrations with sandwich ELISA

HIEC-6 and PHH were incubated with inhibitors MI-1900 or MI-1907 at 50 µM for 24 h. Cells were fixed with 100% methanol (MeOH, Merck, Darmstadt, Germany) for 10 min and stained on the membrane inserts. Then, both of the cells were blocked for 20 min at room temperature in bovine serum albumin (BSA) solution (phosphate-buffered saline (PBS) buffer supplemented with 5% BSA (Merck, Darmstadt, Germany). Sections were incubated for 1 h at room temperature in presence of anti-occludin rabbit polyclonal primary antibody (1:200, Merck, Darmstadt, Germany). The antibodies were previously diluted in 5% BSA solutions. Then, the inserts were incubated with Alexa-Fluor 546-conjugated anti-rabbit IgG secondary antibodies (1:200, Invitrogen, Thermo Fisher Scientific, Waltham, MA), which were diluted in PBS. The sialic acid residues in HIEC-6 and PHH cell membrane were stained with wheat germ agglutinin conjugated with Alexa-Fluor 488 (1:200 diluted in PBS, WGA Alexa Fluor 488, Invitrogen, Thermo Fisher Scientific, Waltham, MA) for 10 min and cell nuclei were stained in blue using 4′,6-diamidino-2-phenylindole (DAPI) (1:500 diluted in PBS, Invitrogen, Thermo Fisher Scientific, Waltham, MA) for additional 10 min. Between incubations, the inserts were washed in PBS for 3 × 5 min. Inserts were fixed on glass slides using fluorescent mounting medium (Dako, Agilent Technologies, Glostrup, Denmark). The occludin localisation was analysed using a Zeiss confocal microscope 63x Plan Apochromat 63x/1.4 Oil DIC M27 (Zeiss LSM 710 Confocal Microscope, Oberkochen, Germany).

To quantify the occludin concentrations in HIEC-6 and PHH, human occludin sandwich ELISA kits were used (Elabscience, Central European Biosystems, Budapest, Hungary). HIEC-6 and PHH were incubated with inhibitors MI-1900 and MI-1907 at 50 µM for 24 h. The cells were dissociated with 0.25% trypsin solution and the cell suspension were collected and centrifuged for 5 min at 1000×*g*. The cells were suspended with pre-cooled PBS, then were centrifuged for 10 min at 1500×*g* at 5 °C. The cell free supernatants were collected and the ELISA tests were carried out according to the manufacturer’s instructions and measured by fluorometer (Victor X2 2030, Perkin Elmer, Waltham, MA) at 450 nm.

### Models of TMPRSS2 in complex with inhibitors MI-1900 and MI-1907

The homology model of TMPRSS2 was generated with SWISS-MODEL (http://swissmodel.expasy.org/)^30^ and is based on the crystal structure of human plasma kallikrein (PDB: 2ANY). For inhibitor MI-1900, the homology model was superimposed with the crystal structure of a structurally related Phe(3-Am)-derived inhibitor in thrombin (PDB: 4E7R^13^). Afterwards, thrombin was deleted and the original inhibitor MI-432 was converted into the structures of analogs MI-1900 and MI-1907 using the builder function of the software Molecular Operating Environment (MOE, version 2019, Chemical Computing Group, Montreal, Canada), followed by an energy minimisation of the complexes using MOE.

### Statistical analysis

For statistical evaluation, R 2.11.1 software package (2010) was applied. Statistical significance of differences was assessed with one-sample Student’s *t*-tests for assessment of relative values. Differences between absolute means were evaluated by one-way analysis of variance (one-way ANOVA) with post hoc Tukey test, where data were of normal distribution and homogeneity of variances was confirmed. Differences were considered significant if the *p* values was <.05 marked with * (***p<.001).

## Results

### MTS assay

**Figure 2.**
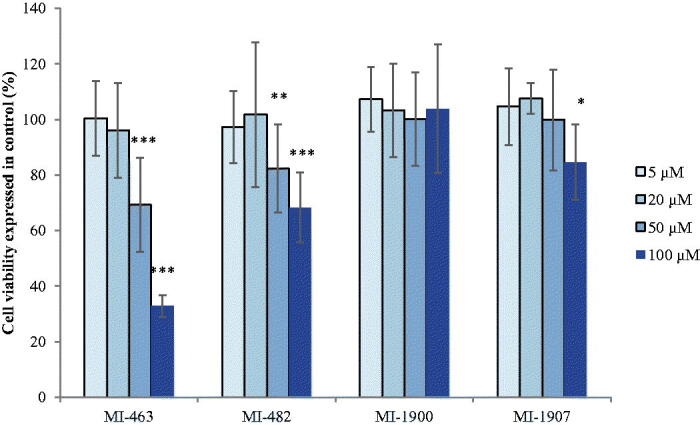
Cell viability assay of the used inhibitors in HIEC-6. The data show the mean absorbance values ± SDs. The treatment lasted for 24 h. *Significant differences in cell death rates in the treated and in the control groups (*p<.05, **p<.01, ***p<.01, *n* = 8).

**Figure 3.**
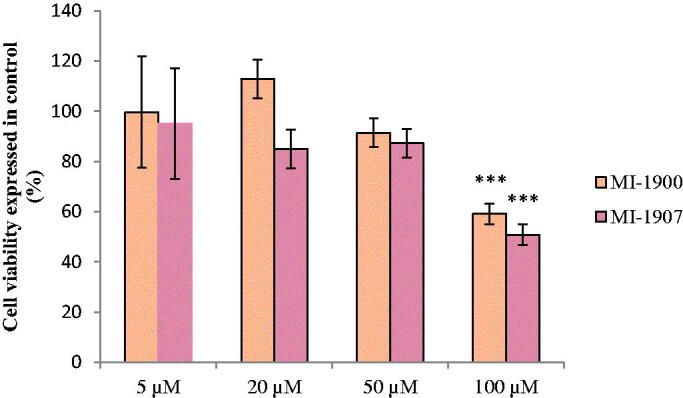
Cell viability assay of the used inhibitors in PHH. The data show the mean absorbance values ± SDs. The treatment lasted for 24 h. ***Significant differences in cell death rates in the treated and in the control groups (***p<.01, *n* = 8).

The MTS assay was used to assess the viability of the HIEC-6 and PHH 24 h after inhibitor treatment. The cells were incubated with each inhibitor at different concentrations such as 5, 20, 50, and 100 µM. The cell viability assay showed that MI-463 and MI-482 treatments elevated HIEC-6 cell death rate at 50 µM and 100 µM (*p*<.001 in case of MI-463 and *p*<.01 and *p*<.001 in case of MI-482, respectively). In addition, MI-1907 also decreased the cell viability of HIEC-6 but only at 100 µM (*p*<.05). However, MI-1900 did not appear to be cytotoxic at any applied concentration after 24 h treatment (Figure 2). Based on this result, PHH were only treated with MI-1900 and MI-1907 for 24 h. Both inhibitors decreased the cell viability of the PHH significantly but also only at 100 µM (*p*<.001) (Figure 3).

### Assessment of extracellular H_2_O_2_ production

**Figure 4.**
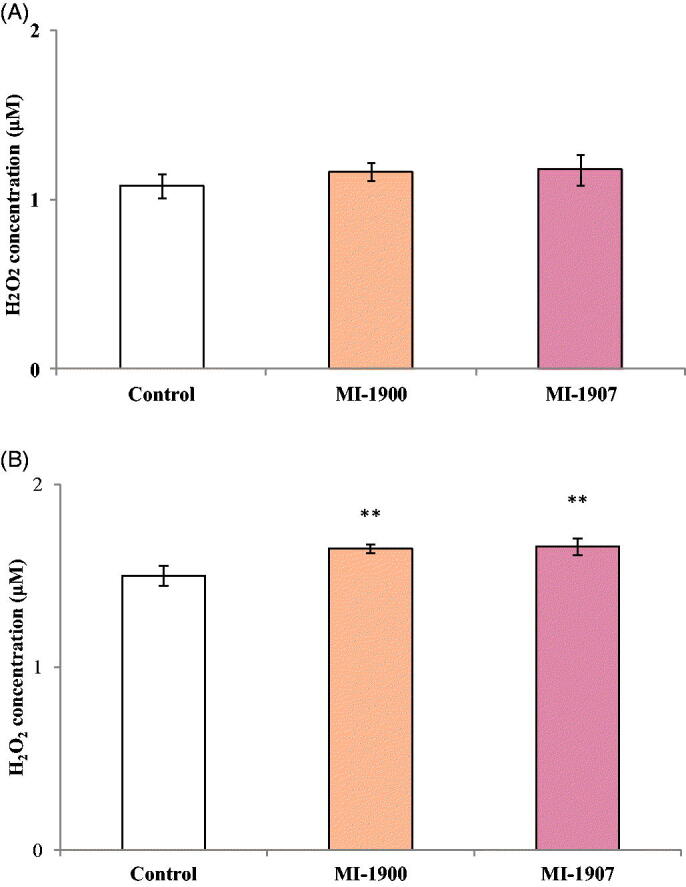
Quantification of extracellular hydrogen peroxide levels after inhibitor treatment (50 μM applied concentrations) using the Amplex Red reagent in HIEC-6 (A) and PHH (B). The data show the mean H_2_O_2_ concentrations (µM)±SDs. The treatment did not cause significant differences of the extracellular H_2_O_2_ production between the control and the treated groups in HIEC-6 (*p*>.05, *n* = 8). On PHH, the treatment elevated the extracellular H_2_O_2_ production, in comparison to the control group (*p*<.05, *n* = 8).

To quantify the H_2_O_2_ production, cells were incubated with the inhibitors MI-1900 and MI-1907 at the largest measured non-cytotoxic (50 µM) concentration for 24 h. After the treatment, cell free supernatants were taken from the basolateral compartment into a 96-well plate and were mixed with the Amplex Red working solution followed by the measurement of the fluorescence intensities. The results show that none of the inhibitors significantly increased the production of the extracellular hydrogen peroxide at the applied concentration (*p*>.05) (Figure 4(A)), thus, extracellular redox balance was maintained in HIEC-6 exposed to the inhibitors for 24 h. However, the administration of MI-1900 or MI-1907 at 50 µM resulted in significant elevations in extracellular H_2_O_2_ production in PHH after 24 h (*p*<.05) (Figure 4(B)).

### Determination of expressions for pro-inflammatory IL-6 and IL-8 cytokines

**Figure 5.**
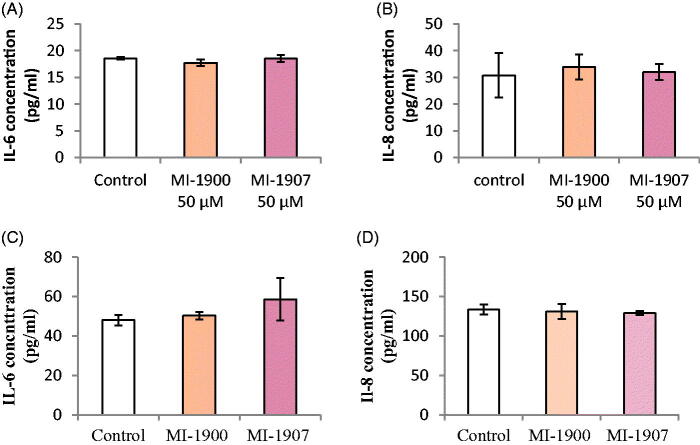
Sandwich ELISA assay for IL-6 (A, C) and IL-8 expression (B, D) after 24 h treatment of HIEC-6 (A, B) and PHH (C, D) with inhibitors MI-1900 or with MI-1907 at 50 µM. The data show the mean IL-6 and IL-8 concentrations (pg/mL ± SDs). None of the inhibitors increased IL-6 and IL-8 concentrations after 24 h treatment (*p*>.05, *n* = 8).

To assess the pro-inflammatory IL-6 and IL-8 levels of HIEC-6 and PHH after 24 h treatment with inhibitors MI-1900 or MI-1907 at a concentration of 50 µM, human IL-6 and IL-8 sandwich ELISA methods were used. At the used concentration, inhibitor MI-1900 and MI-1907 did not cause significant changes in IL-6 (*p*>.05) and IL-8 levels (*p*>.05) in HIEC-6 and PHH (Figure 5(A–D)).

### Assessment of occludin distribution and concentrations in HIEC-6

**Figure 6.**
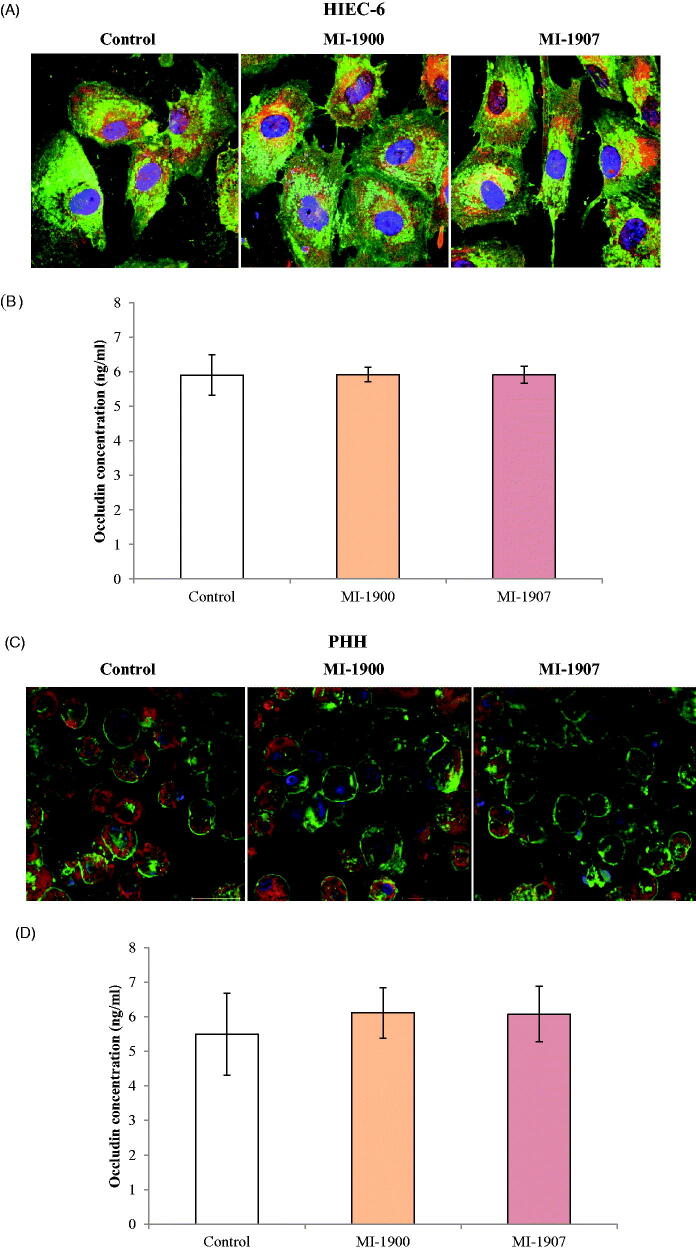
Influence of inhibitors MI-1900 and MI-1907 on occludin expression. (A) An immunofluorescence staining of occludin in controls and in inhibitor-treated cells (50 µM) for 24 hours. HIEC-6 cells were cultured on membrane inserts for eight days and subsequently exposed to inhibitors MI-1900 and MI-1907. Cells were labelled for occludin by incubating them with an anti-occludin rabbit polyclonal primary antibody followed by treatment with Alexa-Fluor 546-conjugated anti-rabbit IgG secondary antibodies. Scale bar shows 20 µm. (B) Quantitative determination of occludin levels after 24 hours treatment of HIEC-6 with inhibitors MI-1900 and MI-1907 at 50 µM. The data show mean occludin concentration values in ng/mL units ± SDs. There were no differences in occludin levels between control and MI-treated HIEC-6 (*p*>.05, *n* = 6). (C) An immunofluorescence staining of occludin in controls and in inhibitor-treated cells (50 µM) for 24 hours. PHH cells were exposed to inhibitors MI-1900 and MI-1907. Cells were labelled for occludin by incubating them with an anti-occludin rabbit polyclonal primary antibody followed by treatment with Alexa-Fluor 546-conjugated anti-rabbit IgG secondary antibodies. Scale bar shows 30 µm. (D) Quantitative determination of occludin levels after 24 hours treatment of PHH with inhibitors MI-1900 and MI-1907 at 50 µM. The data show mean occludin concentration values in ng/mL units ± SDs. There were no differences in occludin levels between control and MI-treated PHH (*p*>.05, *n* = 6).

Localisation of occludin in TJ assembly was assessed in untreated control and in inhibitor-treated HIEC-6 and PHH using immunofluorescence staining. The cells were investigated 24 h after MI-1900 and MI-1907 treatments. It can be seen that the localisation patterns of occludin did not significantly change by continuous inhibitor administration on neither type of cell (Figure 6(A,C)). Occludin concentrations were determined in control and in cells exposed to inhibitors MI-1900 and MI-1907 at 50 µM for 24 h. It was found that basal occludin concentrations did not change significantly after inhibitor treatment (*p*>.05, *n* = 6) in either HIEC-6 or PHH (Figure 6(B,D)).

### Homology model of TMPRSS2 in complex with inhibitors MI-1900 and MI-1907

**Figure 7.**
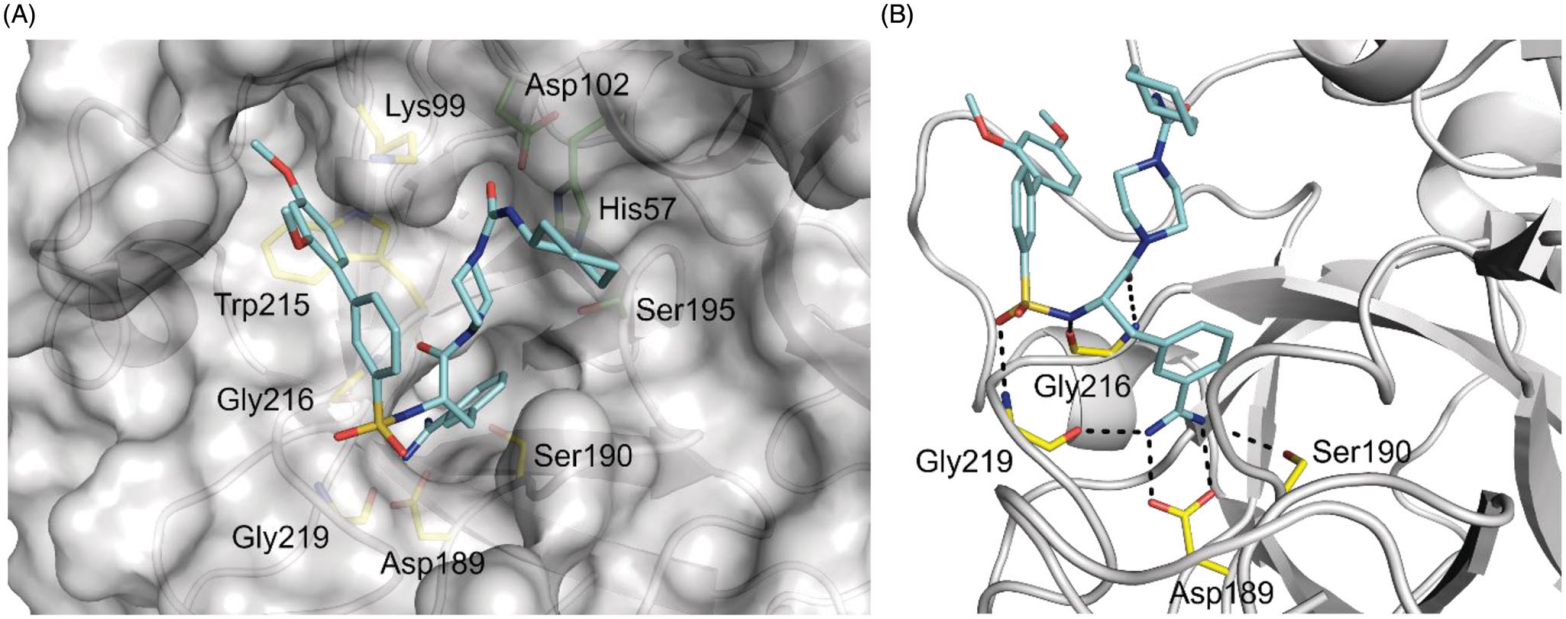
Model of inhibitor MI-1900 (pale blue carbon atoms) in complex with TMPRSS2. (A) View of the inhibitor embedded into the active site of TMPRSS2 shown with a transparent surface. The residues of the catalytic triad (Ser195, His57, Asp102, residue numbers are based on the chymotrypsinogen numbering) are depicted with green carbon atoms, important residues of the active site involved in polar contacts to the inhibitor are shown with yellow carbon atoms. Lys99 is oriented towards the biphenyl’s terminal ring and might form a cation–π interaction. (B) Close view on the active site interactions. The amidino group forms strong ionic interactions with Asp189 at the bottom of the S1 pocket and polar interactions with the surrounding residues Ser190 and Gly219. Furthermore, the sulfonylated backbone makes an antiparallel beta sheet interaction with Gly216 and binds to the NH of Gly219.

**Figure 8.**
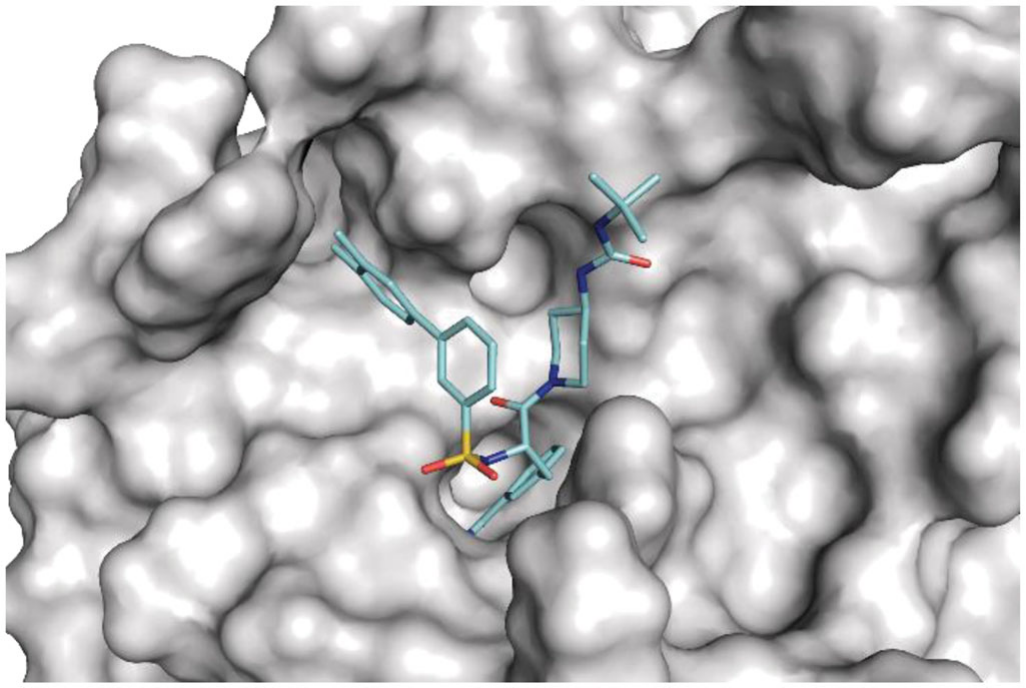
Model of inhibitor MI-1907 (pale blue carbon atoms) in TMPRSS2 shown with its solvent-exposed surface in grey.

Since numerous crystal structures of Phe(3-Am)-derived inhibitors in complex with other trypsin-like serine proteases have been determined in the past, the general binding mode of such inhibitors is well established. Accordingly, known key interactions (Figures 7 and 8) were taken into account in the creation of the modelled inhibitors MI-1900 and MI-1907 in complex with TMPRSS2.

Both compounds adopt a Y-shape conformation typical for such Phe(3-Am)-derived inhibitors in complex with trypsin-like serine proteases. As shown in Figure 7(A,B), the Phe(3-Am) core is deeply bound into the S1 pocket, the biphenyl’s terminal phenyl ring occupies the S3/4 binding pocket above Trp215 and is surrounded by the basic side chain of residue Lys99.

In these models, the ureido piperidide and piperazide, respectively, do not interact with any specific residues of the protein.

## Discussion

TMPRSS2 is highly expressed in lung tissues and in cells derived from subsegmental bronchial branches^31^ and it can be also found in the aerodigestive tract^32^ and in type II alveolar cells and alveolar macrophages^33^ in addition to its presence on the luminal side of the normal prostatic epithelium.

Serine protease activities seem to play a fundamental role in the maintenance and functioning of epithelial barrier homeostasis. It was reported that administration of trypsin and human neutrophil elastase led to increased transepithelial electrical resistance (TER) of confluent layers of airway and alveolar cells and consequently reduced paracellular ion conductance suggesting the physiological role of serine protease in fluid management across lung epithelium^34^.

Intestinal epithelium takes part in protection of the host organism against damaging external xenobiotics. Pancreatic digestive serine proteases including trypsin, chymotrypsin, and elastase could elicit TERs of polarised monolayers for three intestinal epithelial cell lines such as nontumourigenic canine SCBN and human colon adenocarcinoma, Caco-2 and T84. In addition, treatment with these serine proteases increased localisation of occludin into the cell junctional complex presumably mediated by activation of PKCzeta^35^.

Using soybean trypsin inhibitor, it was also proven that trypsin and the transmembrane serine protease matriptase-induced reinforcement of the barrier function manifested in elevated TER was dependent on the catalytic activity of these proteases^36^.

Loss of matriptase resulted in reduction of TER in intestinal tissue segment of suppression of tumourigenicity (*St14*) hypomorphic mice and in Caco-2 cells treated with the broad-spectrum serine protease inhibitor, AEBSF or the selective synthetic matriptase inhibitor CVS-3983. Blocked matriptase activity can influence physiological turnover of tight junctional claudin-2 protein responsible for decreasing the tightness of epithelial barriers. Supportive matriptase-based intestinal barrier recovery can be one of the strategic options in the prevention and in the treatment of intestinal bowel diseases with weakened barrier integrity^37^^,^^38^.

Modulation of TJ assembly was studied in human adenocarcinoma cell line, HT-29 by freeze-fracture electron microscopy and it was found that TJ could be induced by administration of endopeptidases such as trypsin, chymotrypsin, collagenase, elastase, plasmin, and thrombin.

In our previous work, the inhibition of TMPRSS2 in IPEC-J2 cells via administration of the structurally related inhibitor MI-432 was detected at protein level using Western blot analysis. Under denaturing conditions of the electrophoresis the activated serine protease domain (28 kDa) was absent and a decrease in 62 kDa truncated fragment was also observed when the cells were incubated with this inhibitor at 50 µM for 48 h. Decreased tryptic activity was also measured 48 h after treatment with MI-432 in cell supernatants. It was also ascertained that exposure of IPEC-J2 cells to MI-432 at 10, 25, and 50 µM concentrations for 48 h could reduce the membrane presence of TMPRSS2^39^^,^^40^.

Nontumourigenic human intestinal epithelial HIEC-6 cells are capable of reaching confluency and form a monolayer^41^. Detectable TJ proteins such as claudin-1 and occludin were also reported in HIEC-6^42^; therefore, this cell line could be a suitable model for analysing the alterations of the changes in TJ protein assembly and the barrier conditions *in vitro*.

In our study, occludin mapping revealed that distribution pattern and amount of occludin remained the same in HIEC-6 exposed to inhibitors MI-1900 and MI-1907 at 50 µM for 24 h. In contrast, occludin relocalisation was detected in IPEC-J2 cells after treatment with inhibitor MI-432 at 50 µM. Exposure of IPEC-J2 cells to MI-432 and to a second analogue MI-460 revealed that these inhibitors could cause a significant decrease in matriptase activity, which also led to significant reduction of the TER and an enhancement in transport of fluorescently labelled dextran molecules in addition to cellular redistribution of occludin^43^^,^^44^.

Cytotoxicity of few Phe(3-Am)-derived inhibitors has been previously investigated. CU-1804 and CU-1807 (10 µM, 48 h) did not affect viability of human pancreatic adenocarcinoma AsPC-1 cells. The inhibitors MI-432 and MI-462 at 50 µM neither exerted cytotoxic effects on Calu-3 cells during 48 h exposure time^45^^,^^46^.

In our study, the non-cytotoxic concentrations of inhibitors MI-1900 and MI-1907 were determined. It was proven that MI-1900 could be safely administered at concentrations up to 100 µM and had no significant effect on of cell viability of HIEC-6 after 24 h treatment period. MI-1900 and MI-1907 can be tolerated up to 50 µM in PHH similarly to HIEC-6. Even though the primary cell cultures such as PHH have a disadvantage of shorter lifespan and limited availability, these cultures are more likely better suited for pharmacological and toxicological researches because of their greater sensitivity, compared to cell lines, e.g. HepG2 or HeraRG. Hepatic enzyme induction as one of the most essential parameters for pharmacokinetic research is significantly greater in primary hepatocytes than hepatic cell lines^47^^,^^48^.

Tumour necrosis factor alpha (TNFα), IL-6, IL-8 levels were previously studied to elucidate inflammatory responses in HIEC-6 after exposure of IL-17 and lipopolysaccharide (LPS)^49^^,^^50^. ROS and cell viability were also measured by establishing the effects of 3,3′-diindolylmethane (DIM) antioxidant agent on HIEC-6^51^. Using MI-1900 and MI-1907, the extracellular hydrogen-peroxide production and release of pro-inflammatory cytokines such as IL-6 and IL-8 were monitored and it was confirmed that both compounds at 50 µM did not induce excessive oxidative or inflammatory responses in HIEC-6 similarly to other Phe(3-Am)-derived inhibitors such as MI-460 and 461 in hepatocytes-based cell cultures^52^^,^^53^.

It was also proven that MI-1900 and MI-1907 did not generate inflammatory responses in PHH; however, these inhibitors induced oxidative stress at 50 µM. Based on our findings, HIEC-6 appeared to be appropriate *in vitro* model system for mimicking biological processes such as changes in redox homeostasis and inflammatory responses to xenobiotic exposure in human intestine. In accordance, the broad-spectrum inhibitors of numerous trypsin-like serine proteases did not cause enhanced extracellular hydrogen peroxide production even at 50 µM in IPEC-J2 cells^39^.

In previous publications, the inhibition of the replication and spread of certain influenza virus strains (H1N1, H3N2, and H9N2) by Phe(3-Am)-derived inhibitors^25^^,^^46^^,^^54^ such as MI-432 and MI-462 was reported, which most likely act via an inhibition of TMPRSS2, matriptase or an other trypsin-like serine protease.

The impact of several Phe(3-Am)-derived inhibitors was investigated on porcine intestinal epithelial cell line, IPEC-J2, hepatocyte mono- and hepatocyte-Kupffer cell co-cultures. It was found that 50 µM MI-432, 441, 460, and 461 did not exert any detrimental effects on cell viability of hepatocyte–Kupffer cell co-cultures^52^. In addition, a significant increase in hepcidin levels was detected in hepatocyte mono- and hepatocyte-Kupffer cell co-cultures after acute treatments^53^. However, it has recently been reported that targeting only proteolytic activity of matriptase-2 cannot modulate hepcidin expression in clinically relevant extent^55^.

Bestle et al.^16^ found decreased SARS-CoV-2 titres in a dose-dependent manner after treatment with the Phe(3-Am)-derived inhibitors MI-432 and MI-1900, most likely via an inhibition of TMPRSS2. A five times and 25 times reduction in virus titres were obtained using inhibitor MI-1900 and MI-1907 at 50 µM, respectively. Experiments using HEK293 cells cotransfected with pCAGGS-S-Myc-6xHIS and pCAGGS-TMPRSS2 suggested that S cleavage at the S2′ site is only caused by TMPRSS2 and the combination with inhibitors of the proprotein convertase furin such as compound MI-1851, further enhanced the inhibition of the S protein activation at its S1/S2 site, which is activated by furin.

Camostat mesylate was first approved in Japan 2006 for the treatment of chronic pancreatitis due to its inhibitory effects on cholecystokinin, pro-inflammatory cytokines, and serine proteases in human. Based on their activities against TMPRSS2, camostat and a structurally related serine protease inhibitor, nafamostat are presently investigated as off-label administration in the treatment of SARS-CoV-2-infected patients^56–59^.

Numerous of these Phe(3-Am)-derived inhibitors seem to be non-specific and possess a considerable potency against matriptase, thrombin, or factor Xa as well^46^.

It was previously reported that various mono-, di-, and tribasic Phe(3-Am)-derived inhibitors of trypsin-like serine proteases could effectively reduce the infectivity of certain influenza A viruses in Calu-3, in MDCK or in HEK293 cells^25^^,^^46^^,^^54^. Furthermore, inhibitors MI-432 and MI-1900 could significantly decrease SARS-CoV-2 virus titres in a dose-dependent manner in Calu-3 cells^16^. In these studies, MI-1900 exerted a stronger inhibition of SARS-CoV-2 replication at 50 µM with 25–70-fold reduced virus titres compared to MI-432, which provided an approximately 14-fold decrease in virus titres. At present, it is difficult to define the most promising inhibitor of the Phe(3-Am)-type, because different trypsin-like serine proteases are involved in the glycoprotein activation of various enveloped viruses. However, compared to the tribasic or dibasic analogues, a less polar monobasic Phe(3-Am)-derived inhibitor MI-1900 might be advantageous compound for further studies due to its improved bioavailability. Furthermore, our data suggest that inhibitor MI-1900 could be a safely applicable drug candidate against certain influenza virus strains and SARS-CoV-2.

Phe(3-Am)-derived inhibitors, which may offer promising preventive or therapeutical strategy for treatment of COVID-19 via suppression of various host cell proteases essential for viral entry and replication, was applied and characterised in this study using human intestinal epithelial cells.

## Conclusions

In conclusion, structurally related inhibitors of trypsin-like serine proteases were tested in HIEC-6 and PHH to determine their *in vitro* safety. Recently, in case of MI-1900 an anti-SARS-CoV-2 effect could be demonstrated in Calu-3 cells inoculated with SARS-CoV-2 at a low MOI of 0.001 based on viral titres in cell supernatants. It was also demonstrated that two of the selected Phe(3-Am)-derived inhibitors can be applied safely at concentrations up to 50 µM without affecting occludin localisation pattern and perturbing the regulation of investigated cytokines such as IL-6 and Il-8 levels.
